# Effects of surgical masks on aerosol dispersion in professional singing

**DOI:** 10.1038/s41370-021-00385-7

**Published:** 2021-10-05

**Authors:** Stefan Kniesburges, Patrick Schlegel, Gregor Peters, Caroline Westphalen, Bernhard Jakubaß, Reinhard Veltrup, Andreas M. Kist, Michael Döllinger, Sophia Gantner, Liudmila Kuranova, Tobias Benthaus, Marion Semmler, Matthias Echternach

**Affiliations:** 1 Division of Phoniatrics and Pediatric Audiology at the Department of Otorhinolaryngology, Head and Neck Surgery, University Hospital Erlangen, Friedrich–Alexander-University Erlangen–Nürnberg, Erlangen, Germany; 2 Department of Head and Neck Surgery, David Geffen School of Medicine at the University of California Los Angeles, Los Angeles, CA USA; 3 Division of Phoniatrics and Pediatric Audiology, Department of Otorhinolaryngology, University Hospital, LMU Munich, Munich, Germany; 4 Department of Artificial Intelligence in Biomedical Engineering, Friedrich–Alexander-University Erlangen–Nürnberg, Erlangen, Germany; 5 Institute and Clinic for Occupational, Social and Environmental Medicine, University Hospital, LMU Munich, Munich, Germany

**Keywords:** Aerosol dispersion, Choir singing, Surgical mask, Airborne virus transmission, SARC-CoV-2 pandemic

## Abstract

**Background:**

In the CoVID-19 pandemic, singing came into focus as a high-risk activity for the infection with airborne viruses and was therefore forbidden by many governmental administrations.

**Objective:**

The aim of this study is to investigate the effectiveness of surgical masks regarding the spatial and temporal dispersion of aerosol and droplets during professional singing.

**Methods:**

Ten professional singers performed a passage of the Ludwig van Beethoven’s “Ode of Joy” in two experimental setups—each with and without surgical masks. First, they sang with previously inhaled vapor of e-cigarettes. The emitted cloud was recorded by three cameras to measure its dispersion dynamics. Secondly, the naturally expelled larger droplets were illuminated by a laser light sheet and recorded by a high-speed camera.

**Results:**

The exhaled vapor aerosols were decelerated and deflected by the mask and stayed in the singer’s near-field around and above their heads. In contrast, without mask, the aerosols spread widely reaching distances up to 1.3 m. The larger droplets were reduced by up to 86% with a surgical mask worn.

**Significance:**

The study shows that surgical masks display an effective tool to reduce the range of aerosol dispersion during singing. In combination with an appropriate aeration strategy for aerosol removal, choir singers could be positioned in a more compact assembly without contaminating neighboring singers all singers.

## Introduction

Person-to-person transmission of CoVID-19 mainly proceeds by direct contact or transmission of droplets with diameters ≥5 μm and smaller aerosol particles with diameters ≤5 μm [1] within a range of 0.001–500 μm [2, 3]. Whereas virus-laden droplets are only received when a person is in close proximity to the emitter, aerosol particles remain hovering in the air and convectively spread in the environment distributing within large regions and rooms. Therefore, the infection with SARS-CoV-2 can potentially take place via reception of expelled droplets and aerosol particles from an infected person, or by inhalation of accumulated aerosol particles in closed rooms without appropriate ventilation.

According to the super-emitter theory [4], the expulsion of a much larger amount of droplets and aerosol particles owing to exhalation activities such as extensive and loud speaking, singing, sneezing, or coughing poses the highest risk for CoVID-19 transmission. Especially singing turned out to be a high-risk activity for aerosol and droplet production [5–7] resulting in fatal infectious events during choir singing as documented in Amsterdam, Berlin, and Skagit Country (WA, USA) [8–10].

Speaking is thought to produce about three times the absolute amount of aerosol compared to breathing [11] and the particle emission rate during different spoken text passages increases for phrases that include a higher number of vowels [12]. Singing with its exaggerated articulation, as defined by the sustained vocalization of a vowel, is associated with a much higher rate of aerosol emission, showing ten times higher values than breathing and about three times higher values than speaking [11]. Furthermore, the exaggerated articulation produces a larger amount of ballistic droplets. In recent studies, an even higher production of aerosol was observed with a 4 to 100 times greater amount produced by singing compared to speaking [13–15]. Thereby, the directly expelled aerosol cloud has the highest density of potentially virus-laden aerosol particles and ballistic droplets constituting a high risk of infection for people staying in that region. This region is located directly in front of the singer up to a distance of 1.5 m to front and 1 m to the sides [16]. As a consequence, in scientific and administrative evaluations, singing has become associated with a much higher risk for person-to-person transmission of infectious diseases, such as CoVID-19. Consequently, in certain countries across the globe, singing in choirs and at religious services was restricted or prohibited [17, 18] because especially in choirs, the singers stay in close proximity to each other which is essential for the quality of the performance.

Beside social distancing, wearing masks and face shields are the most effective arrangements to lower the risk of airborne transmission of viruses such as Influenza, SARS-CoV, MERS-CoV, and SARS-CoV-2 [19, 20]. In fact, aerosol particles and ballistic droplets were found to be reduced if the emitter wore a mask [21] and overall transmission could further be reduced if both the emitter and receiver wore masks [22] especially for surgical and KN95 mask types. However, leakages at the cheeks and nose bridge were found in experimental [23, 24] as well as in numerical studies [25, 26] through which especially aerosol particles escape with the leakage flow. In contrast, the droplets with ballistic behavior especially those generated by coughing and sneezing are effectively filtered out [27] as long as the face mask is composed of three layers as common medical masks (surgical, N95, KN95) [28].

With regard to droplets, the distance from the mouth depends on many factors, such as loudness [29, 30] or the type of activity such as just vocalizing or speaking especially by articulating consonants. For coughing, droplets have been detected at distances up to 8 m from the mouth [29]. After being expelled, these droplets follow ballistic trajectories and descend to the ground quickly [30–32]. For the investigation of the dispersion of expelled aerosol particles and droplets, qualitative, and quantitative flow visualization techniques as smoke visualization with bright light or laser light-sheet illumination, Particle Image Velocimetry [33] (PIV, with derivates as Particle Tracking Velocimetry) or Schlieren visualization [34, 35] have been applied using mostly manikin models [35, 36] or single human subjects [37, 38].

Although singing turned out to be highly critical [5–7], the respiratory activities that were mainly studied are breathing, coughing, sneezing, and speaking. Furthermore, the distances reached by the aerosol particles and droplets were only measured in the main expulsion direction [23, 39] without taking into account all three spatial directions.

Thus, the aim of this study is to analyze the spatial and temporal dispersion of aerosol particles during professional singing with a surgical mask in comparison with singing without masks. In a second experiment, we detected ballistic droplets in front of the singer with and without mask to prove the filtering efficiency. The experiments were performed in a studio of the Bayerischer Rundfunk (BR: Bavarian public-service radio and television broadcaster) with ten professional singers of the BR choir (five males and five females) serving as subjects. With the study, we contribute data to answer the question were the region with the highest aerosol concentration is localized and whether and how a surgical mask influences the dispersion of expelled aerosol particles and the ballistic droplet during professional singing.

The goal is how the application of these masks could contribute to develop strategies to maintain and support choir rehearsals and performances during an airborne spreading virus pandemic as CoVID-19.

## Methods

After approval from the local ethics committee (20–395), ten professional full-time singers from the BR Choir, all nonsmokers and without pulmonary symptoms, were included in the study (five female, five male, age 44 ± 11 years). All subjects practice western classical singing and had completed their vocal studies at music conservatories. No subject complained of dysphony: normal values (mean 2 ± 3) were obtained for a German version of the singing-voice-handicap-index-12 [40].

### Singing tasks

All subjects were asked to perform a part of the melody from the fourth movement “Ode of Joy” of Ludwig van Beethoven’s 9th symphony to the original text, Freude schöner Götterfunken, Tochter aus Elysium” written by Friedrich Schiller, in the key of D major, thus starting on F#3 for the male (fundamental frequency f_0 ≈ 185 Hz) and F#4 (f_0 ≈ 370 Hz) for female voices, respectively. The duration of singing task lasted approximately 6–10 s depending on the individual speed of the single singer. In a second task, the singers were asked to perform the task again while wearing a standard surgical mask. During the experiments, the singers were frequently encouraged to drink water in a comfortable amount.

### Aerosol visualization

All measurements were performed in a studio of the BR with the approximate dimensions: 27 m × 22 m × 9 m (width × length × height). The singer stood on a lifting platform to lift them to the correct height. A mark at the singer’s forehead was used as a point of reference to adjust the height of the platform to the correct position. The wall was 4 m behind and 5 m to the left of the platform. To the right and in front of the platform, the walls were more than 7 m away. Three measuring rods for each spatial direction were mounted onto the lifting platform to convert the pixel dimension of the recorded pictures into metric dimensions. The rods were assembled in a cross configuration and had a 0.02 m and 0.1 m scale, respectively. A picture of the setup is shown in Gantner et al. [41].

Three full HD Sony HDC 1700R television cameras (Sony, Tokio, Japan) (resolution 1920 × 1080 pixels) recorded the singers and the cloud from a side view (camera 1), a front view (camera 2) and a top view (camera 3) perspective. The side and front camera were equipped with Canon DIGI SUPER 25 XS lenses (Canon, Tokio, Japan) and the top camera with a HD Fujinon HA14x4.5BERM/BERD wide angle HD lens (Fujinon, Tokio, Japan). All cameras recorded synchronously at a frame rate of 25 fps. To detect the start and end of singing, the audio signal was recorded with a Sennheiser KMR 81 directional and a ME62 omnidirectional microphone (Sennheiser electronic GmbH, Wedemark, Germany) which was placed at a corner of the singer’s lifting platform, in 1.5 m distance to the mouth.

To visualize the expelled air during singing, all subjects inhaled the vapor of a Lynden Vox e-cigarette (Lynden GmbH, Berlin, Germany) filled only with the basic liquid, which consists of 50% glycerin and 50% propylene glycol as similarly applied in [23, 24, 33, 42, 43]. According to Ingebrethsen et al. [44] the particles generated in e-cigarettes have a diameter in the range of aerosol particles at 250–450 nm. For each task, the singer’s individual inhaling volume was measured by a ZAN 100 spirometer (Inspire, Oberthulba, Germany) which was coupled with the mouthpiece of the e-cigarette. Being already on the platform, each singer was asked to inhale 0.5l vapor controlled by the spirometer. Immediately after inhalation, the singers moved to the singer’s mark and started to perform the task. The singers were instructed to maintain their position on the platform without moving for up to 60s after completing each task, to allow the exhaled cloud to be traced. Because all the singers were nonsmokers, the inhalation was tested by the subjects before the experiments to become accustomed to the vapor. In rare cases of sudden coughing during the experiments, the task was repeated until it was performed correctly.

In order to achieve a large visual contrast between the cloud of vapor and the background, the entire studio was lined with black linen and the singers wore black clothes. The vapor was illuminated with three flash lights positioned on the left-hand side of the platform: one in the left corner behind the platform with a distance of ~5 m to the singer, one in front of the platform on the left side in a distance of 7 m and another directly behind the platform ~3.5 m from the rear edge of the platform.

Before each task, the studio was aerated with the main gate open for at least 2 min using a fan behind the platform. The main gate was at the opposite side of the studio with a distance of ~20 m to the platform. Afterwards, the gate was closed and everyone present was instructed to stop moving for another 2 min to settle down air circulation that remained from the active ventilation. Subsequently, the task started with the singer’s inhalation of the e-cigarette basic liquid. The temperature and the humidity in the studio were measured and recorded. The temperature was measured at mean 23.27 °C (SD 0.46 °C) and the relative humidity at 46.12% (SD 0.95 °C), respectively.

### Droplet visualization

**Fig. 1:**
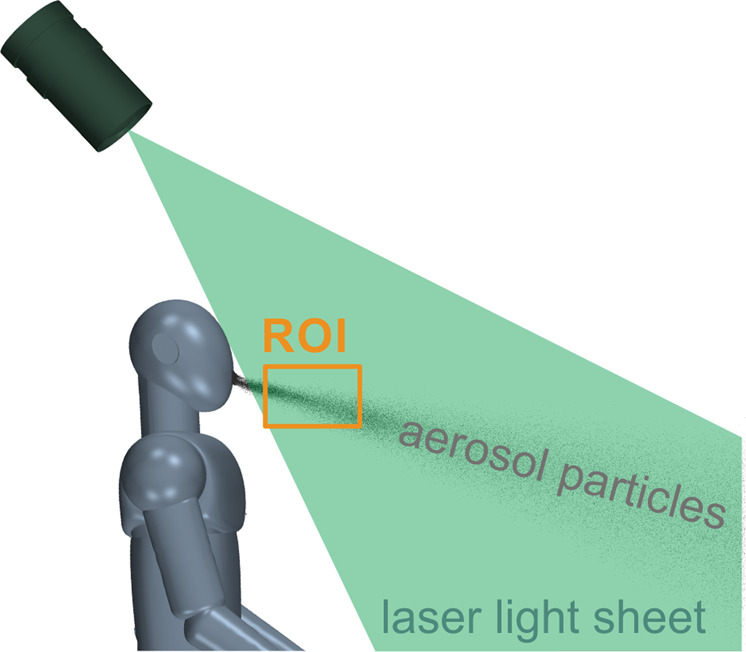
Principle of the droplet visualization with a laser light sheet placed in front of the singer. The particles were recorded by a highspeed camera within the region of interest (ROI).

For the visualization of the ballistic droplets in the diameter range larger than 5 μm [1], the singer performed the two tasks with and without mask within a dark chamber that was also installed in the BR television studio. Similar to the study of speaking and coughing in [45], the droplets were illuminated by a laser light sheet that was positioned in a sagittal orientation immediately in front of the singer subject as shown in Fig. 1. The laser beam was produced by a Coherent Titan-Sapphire Verdi V6 laser (*λ* = 532 nm) with a maximum output power of 6W (Coherent Inc. Corporate, Santa Clara, CA, USA). The circular laser beam was guided through an ILA mirror arm (iLA5150 GmbH, Aachen, Germany) and transformed into a 0.002 m thick light sheet by a cylindrical lens with a divergence angle of 30°. The exit opening of the mirror arm was positioned above the singer’s head and emitted the laser light-sheet downwards. During the experiments, the laser was operated at an output power of 3 W. The singer subjects were not directly exposed to the laser light and wore appropriate laser safety glasses.

The particles were recorded by a Phantom v2511 high-speed camera (Vision Research Inc., Wayne, NJ) within a region of interest (ROI) that was located directly in front of the singer’s mouth. The frame rate amounted to 2000 Hz. The dimensions of the ROI amounted to ROI_*x*_ × ROI_*z*_ = 48 × 76 mm^2^ which was resolved by 1280 × 800 pixels yielding a pixel resolution of ~60 μm/pixels which constitutes a similar resolution as applied by [45] for speaking and coughing and commonly used in PIV measurements [33, 46] classically using tracer particles in the range of 1–5 μm in diameter. Thus, the setup enabled to detect particles with diameters down to 10 μm although the actual size cannot be determined for droplets being smaller than resolution of 60 μm/pixel. The distance between the camera and the laser light sheet was ~0.7 m. Similar to the test setup for the aerosol cloud recording, the singer was elevated to the correct positions by the lifting platform.

### Segmentation of aerosol cloud

**Fig. 2:**
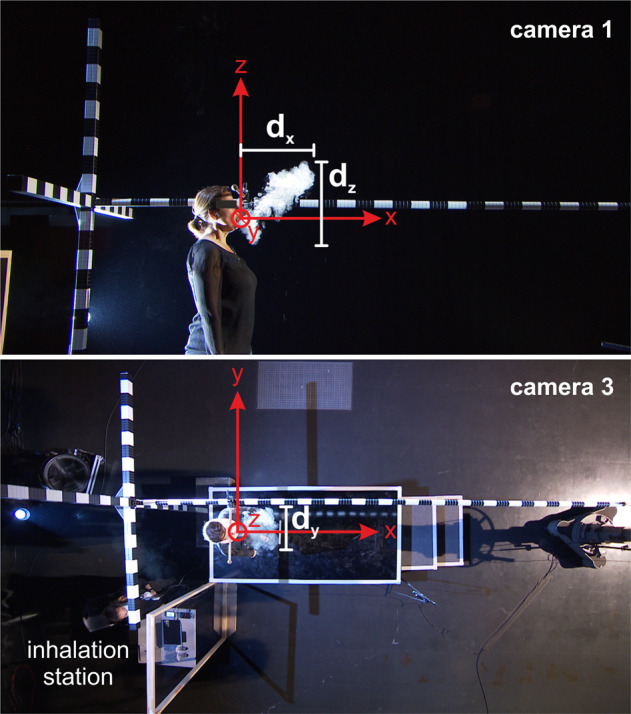
Pictures of camera 1 (top) and camera 3 (bottom) views with the defined coordinate system. Based on the segmentation of the cloud, its maximum dimensions *d*_*x*_, *d*_*y*_, and *d*_*z*_ were computed in each frame.

A coordinate system was defined to segment the video footage in three directions with the origin at singer’s the mouth with *x*-direction to the front, *y*-direction transversely from the left to the right, and *z*-direction vertically from the bottom to the top, as shown in Fig. 2. To evaluate the temporal evolution of the exhaled vapor, the video footage of cameras 1 and 3 was converted into grayscale and the singer was covered with a black mask to avoid the segmentation of bright features such as skin or hair. The aerosol cloud was then segmented in each video frame which was performed with an in-house software tool using a threshold-based region-growing algorithm yielding the area of the cloud and its contour as a function of time. The dimensions of the ROI are ROI_*x*_ × ROI_*y*_ × ROI_*z*_ = 260 cm × 270 cm × 180 cm for camera 1 and 190 cm × 270 cm × 180 cm for camera 3. Based on the cloud contour, the maximum dimensions of the cloud) (*d*_*x*_*, d*_*y*_*, d*_*z*_) as indicated in Fig. 2 in each frame were computed. Thus, the *d*_*x*_ and *d*_*z*_ dimension were calculated from side view (camera 1) and *d*_*y*_ from top view (camera 3).

Based on the segmented aerosol cloud, its dispersion was computed in each spatial direction. Several outliers occurred when non-vapor regions were segmented if the cloud reached a region with similar grayscale value as e.g., the white sectors of the measuring rods or remaining areas of bright skin of the singer’s face, neck, and arms. Those outliers were removed by a moving median filter with a fixed window length of 30 time points and subsequent cubic spline approximation performed in Matlab (The MathWorks Inc., Natick, MA, USA). More details about the post-processing procedure are shown in Westphalen et al. [47].

### Droplet particle detection and tracking

The laser-illuminated particles were tracked by an in-house software tool written in Python. In a first step, all particles were detected in each frame based on a grayscale threshold method. In a second step, tracking was initialized by assigning an identity marker to all particles in a frame. For each particle, a distance matrix was calculated to allocate those particles in the consecutive frame that have a distance of maximum 50 pixels. Subsequently, a deviation score was calculated for each allocated particle based on parameters as particle size, direction of motion and velocity. The particle with the lowest deviation score is then assigned as being identical with the particle of the preceding frame. Furthermore, this algorithm also allows a temporarily disappearance of particles if particles are moving in the boundary regions of the laser light sheet with lower laser intensity.

Owing to the environmental conditions in the television studio, the air was not free from dust particles which were also illuminated by the laser light sheet. Thus, a post-processing step of the identified particle data was performed to exclude those particles that did not originated from the singer. The following rules were defined to identify potential dust particles that hover in the air not originating from the singer’s respiratory tract:

- The particles have to occur in at least 11 frames.
- The particles have to move at least 1 pixel between the first and last frame of occurrence.
- The mean velocity of a particle has to be at least 2 pixels per frame.

The post-processing was performed in Matlab (The MathWorks Inc., Natick, MA, USA) and the statistics in IBM SPSS version 24 (IBM, Armonk, NY, USA).

## Results

### Aerosol cloud visualization

**Fig. 3:**
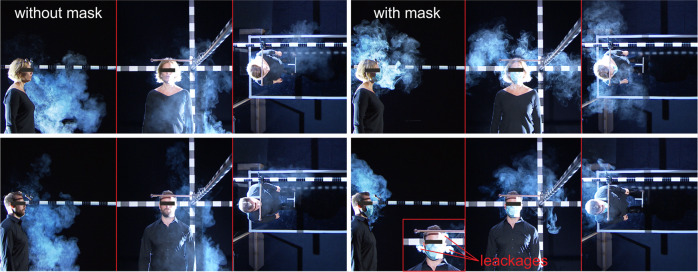
Visualization of the expelled aerosol cloud for two singers in the side, front and top perspective at the end of the singing task. The pictures show singing without (left) and with surgical mask (right). Additionally, a close up in frontal perspective shows the aerosol leakage from the mask at the cheeks and nose. See all singers in Supplementary Figs. 1, 2.

Figure 3 exemplarily shows two singers who emitted the inhaled aerosol cloud during singing with and without surgical masks (see all singers in Supplementary Figs. 1, 2). During singing without mask, the aerosol cloud is majorly expelled into the region in front of the singer with a forward-downward direction of motion. In contrast with a mask, the cloud is located around the singer’s head extended in majorly upward (see side view) and both transverse directions as shown in front and top view at the end of the task. The reason for the different dispersion pattern is that the aerosol cloud escaped through mask leakages at the cheeks and the nose as Fig. 3 shows. As a result, the cloud extent in x-direction is reduced compared to singing without mask.

### Temporal devolution of aerosol cloud dispersion

After the segmentation of the aerosol cloud in the video footage, the temporal devolution of the cloud dispersion was determined in *x*- (to the front), *y*- (transversely to the sides), and *z*-direction (vertically upright) according to the coordinate system shown in Fig. 2. The quantitative dimensions of the aerosol cloud in all directions are shown for the time point 0s (end of task) and 10s after the end of task in Table 1.

**Table 1:** Expansion of the aerosol cloud at (**a**) time point 0 s (end of task) and (**b**) time point 10 s after according to the spatial dimensions introduced in Fig. 2.

**(a)**									
**Time point**	***d***_***x***_**/m**			***d***_***y***_**/m**			***d***_***z***_**/m**		
***t*** = 0 s	**Median**	**Min**	**Max**	**Median**	**Min**	**Max**	**Median**	**Min**	**Max**
Without mask	0.85	0.63	1.28	0.66	0.32	1.12	1.06	0.83	1.29
With mask	0.37	0.15	0.78	1.24	0.64	1.69	0.69	0.43	1.08
**(b)**									
**time point**	***d***_**x**_**/m**			***d***_**y**_**/m**			***d***_z_**/m**		
***t*** = 10 s	**Median**	**Min**	**Max**	**Median**	**Min**	**Max**	**Median**	**Min**	**Max**
Without mask	1.11	0.61	1.39	1.20	0.65	1.98	1.21	0.91	1.38
With mask	0.43	0.00	1.06	1.45	0.05	1.82	0.91	0.005	1.53

The dimensions correspond to the diameter of the aerosol cloud in the basic direction introduced in Fig. 2: *d*_*x*_ axial diameter to the front (*x*-direction), *d*_*y*_ transverse diameter to the sides (*y*-direction), and *d*_*z*_ vertical diameter in upright (*z*-)direction. All dimensions in m.

**Fig. 4:**
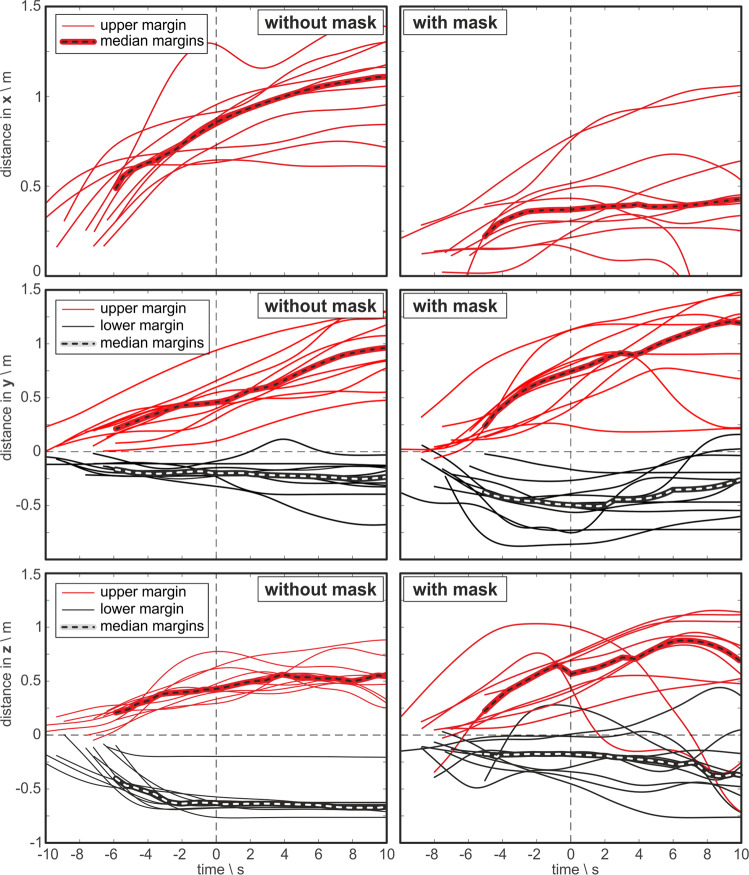
From top to bottom: temporal devolution of the aerosol cloud margin to the front (*x*-direction), in transverse (*y*-), and in vertically upright (*z*-) direction for singing without (left) and with surgical mask (right). The time point 0 s corresponds to the end of the singing task. In accordance to the origin of the coordinate system displayed in Fig. 2, the singer’s mouth is located at *x* = 0 m.

In the top row of Fig. 4, the temporal aerosol cloud dispersion to the front *d*_*x*_ is shown for all singers with and without surgical mask. The median devolution indicates the median cloud expansion over all singers at every time point. The singers’ individual curves reveal a smaller expansion of aerosol cloud to the front *d*_*x*_ when the singers wore a mask. This is also shown by the two median curves representing the two tasks. Moreover, the further expansion of the cloud at the time point 10 s after the end of task is also smaller if a mask was worn. However, there are large individual differences between the individual singers in both tasks. Even two singers reached a maximum distance to the front with masks that is slightly smaller than the median distance for singing without mask at the end of task. In the further progress at 10 s after the end of task, there is a slight increase in median *d*_*x*_ for both tasks as shown in Table 1.

Regarding the transverse direction to the sides, the diameter of the cloud *d*_*y*_ was found to be larger with mask at the end of task as depicted in Fig. 4 middle row. Note here, that the diameter *d*_*y*_ is the difference between the upper (red curves) and the lower margin (black curves) for each individual singer. In the further progression to time point 10s after the end of task, the diameter *d*_*y*_ almost doubled for the task with the mask worn, whereas without mask, the diameter only slightly increased as shown in Table 1.

Another trend could be observed for the cloud expansion in vertical upright (*z*-) direction as shown in Fig. 4 bottom row. In contrast to the transverse direction, the cloud diameter in vertical upright direction *d*_*z*_ decreases as shown in Table 1 if a mask was worn. Furthermore, the cloud moved in upward direction reaching higher levels up to *z* = 1 m above the singer’s mouth compared to *z* = 0.77 m for singing without mask. Similar to the further progression to the front *d*_*x*_, only a slight increase in median *d*_*z*_ occurred for singing with and without mask.

For all three directions, there are individual devolutions which decrease in distance after the corresponding singer has stopped singing at time point 0, as shown in Fig. 4. This decrease is caused by vanishing of the vapor at the borders of the cloud due to dilution and evaporation. As the visibility of the vapor cloud is a function of the density of aerosol particles, the decrease in distance caused an uncertainty in the exact location of the border. However, during the relevant period of active singing until time point 0, this uncertainty is estimated to be small owing to the high contrast between cloud and no cloud regions.

### Droplet particle expulsion

In a second experiment, the ballistic droplets were detected within a laser light sheet and subsequently tracked in the region in front of the singer’s head. It has to be mentioned here that although potential dust particles were excluded from the analysis of the high-speed video footage as described above, it cannot be guaranteed that dust particles were completely excluded. Thus, the results show the relative difference of counted particles between the singing task with and without mask.

**Fig. 5:**
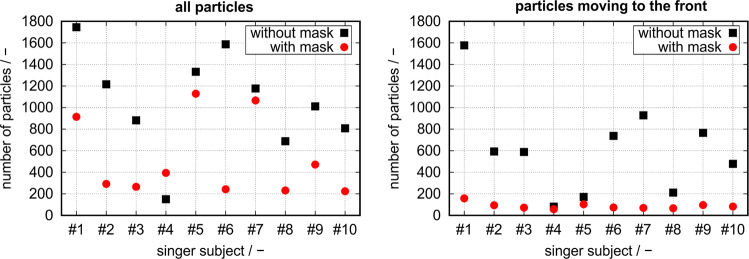
Total number of droplet particles (left) and number of those moving to the front in positive x-direction (right). For both cases, the differences between singing with and without mask were statistically significant tested by the Wilcoxon rank test: total number of particles *z* = −2.497, *p* = 0.013, *n* = 10, number of particles moving to the front *z* = −2.803, *p* = 0.005, *n* = 10.

Figure 5 shows the total number of droplets tracked (left diagram) and the number of those particles which moved exclusively to the front (right diagram). The total number of droplets was found to be significantly reduced by on average 47% for singing with a mask in comparison to singing without mask (Wilcoxon rank test *z* = −2.497, *p* = 0.013, *n* = 10). Furthermore, the number of particles fluctuated for both singing cases, especially for the task singing with mask with singer #4 even more particles than singing without mask. If considering only particles which were emitted through the mouth and therefore assuming that they moved to the front away from the singer, the reduction of particles for singing with mask even significantly increased on average to 86% (Wilcoxon rank test *z* = −2.803, *p* = 0.005, *n* = 10). Furthermore, the number of particles less fluctuates and for eight of the ten singers the number was <100 particles. Again for singing without mask, the fluctuation of the number of particles that only moved to the front (in positive *x*-direction) is much larger.

## Discussion

The aerosol particles and ballistic droplets are mostly generated during breathing, talking, coughing and sneezing [11, 48]. Thereby, coughing and vocalization produce a much higher amount of particles than breathing [11]. Recently, several studies additional analyzed the aerosol particle generation during the sustained phonation or singing [13–15, 49] and found an enhancement of aerosol production by the 3-fold to 300-fold compared to breathing. As consequence, the aerosol particles and droplet expulsion during singing states a high risk to transmit airborne viruses and resulted in several outbreaks of CoVID-19 worldwide [8–10]. All these studies measured the particle count or concentration and did not account for the local distribution of the aerosol particles around the emitter. However, for choir singing this information is critical to define the distances between the singers and between the choir and the audience. Echternach et al. [16] showed that the expelled and highly concentrated aerosol clouds are able to reach distances up to 1.3 m achieved by professional singers. Thus, face masks play an important role not only in relation of their filtering properties but also with regard to their ability to decelerate and deflect the primarily expelled aerosol cloud as previously shown for coughing [50]. The number of studies about the effectiveness of face masks increased since the COVID-19 pandemic has started in late 2019 [20, 51]. These studies show, that face masks are an effective tool to disturb the transmission of an airborne virus by aerosol particles (size 0.1–10 μm) and larger droplets (size ≥10 μm). The number of transmitted viruses could be reduced by 50 and 90% depending on the type of face masks and who is wearing the mask, the emitter and/or the receiver [22].

In our experiments with a laser sheet technique, the amount of tracked droplets was reduced by 47% in this ROI if the singer wore a surgical mask. Especially the reduction of droplets moving in the ejection direction was reduced by 86% on average. Those droplets behave as ballistic particles mainly influenced by their momentum, their initial direction and the gravity force [27, 33, 52]. Similar reduction values were observed for surgical masks for the entire size range of particles (aerosol particles and droplets) [12, 53, 54] although these studies only investigated breathing, speaking and coughing.

Kolewe et al. [53] confirmed in general the effective filtering capabilities of masks. However, they also found a large portion of the expelled particles escaped through leakages at the sides (cheeks) being not filtered out by the mask.

These leakages are critical for the smaller aerosol particles which possess a diameter in the range of 0.3–10.0 μm [5, 6, 37] and thus, hover and follow the air flow in the environment. Similar observations were reported for breathing, coughing and sneezing by using human subjects [24, 33, 50], manikin model [22, 23, 39, 53] or CFD models [26]. Furthermore, leakages were also found for KN95 or N95 masks [25, 42, 55, 56]. Especially in closed rooms without effective ventilation, exhaled aerosol particles can accumulate and develop a critical concentration if single persons are infected.

In this context, our measurements showed that surgical masks reduce the primary momentum and deflect the direction of expulsion as similarly reported for coughing [50]. Thus, at the end of the singing task, the aerosol particles were majorly located in the near-field above and around the singer’s head. In contrast, singing without mask produces an aerosol cloud spreading in front of the singer with far higher distances. Furthermore, the aerosol particles are shifted majorly to higher regions around and above the head with a median distance of ~0.5 m in all directions. This basic principle seems to also hold for other types of masks (community, silk cloth scarfs, FFP2) as Westphalen et al. [47] showed in single subject experiments. Additionally, the aerosol particles being in this near field of the emitter showed a tendency to move further upwards owing to the thermic convection flow due to the in general higher temperature of the emitting person compared to the environmental temperature [36, 57].

In large meeting rooms or e.g., concert halls which are critical regarding infectious scenarios, this condition of the thermal upward drift of the aerosol [36] becomes important because those rooms are mostly equipped with air conditioning and ventilation systems. The ventilation outlets are commonly placed at the ceiling to remove the used air with a high concentration of *CO*_2_ generating a vertical upward flow direction of the expelled aerosol.

The typical surgical mask is not optimized for professional singing. Whereas the usage of surgical masks and typical community masks constitutes an option for layman singers and choirs, the masks might become less effective and hindering due to the exaggerated articulation in professional singing. Hence, special masks for singing should enable large movements of the mouth without increased leakage openings by possibly less influencing the acoustic signal.

### Limitations

The aerosol cloud consisted of the vapor generated from the basic liquid of e-cigarettes. In contrast to the naturally generated and emitted aerosol particles, the vapor consists of a much larger amount of aerosol particles and is, thus, visible. As the contour of the cloud was determined with a threshold-based segmentation algorithm, the exact dimensions of the cloud rely on the intensity of the illumination of the particles and the accuracy of the light-sensitive sensor in the camera. As a consequence, this visualization technique does not allow the detection of aerosol particles in regions where the cloud vanished due to evaporation and/or dilution. For this purpose, other measuring techniques based on particle counting [5–7] have to be applied which, however, deliver the particle count and size only at specific point in the room and thus are not applicable to acquire the spatial and temporal dynamics of an aerosol cloud.

Another limitation of this study is the restricted reproducibility owing to the human singer subjects with their individual expulsion flow characteristics and the unforeseeable nature of unconfined flows. Although the measurements were performed with 10 singers under a strict measuring procedure as described above, differences of the singers’ individual expulsion characteristics cannot be excluded.

In the second experiment, only the relative droplet reduction could be evaluated because the measurements were also performed in the television studies that did not provide a filtering system for the environmental air. Thus, as we mentioned above, dust particles in the air were also recorded which did not originate from the singer’s respiratory tract. As only droplets could be detected and tracked, that were illuminated by the thin laser light sheet in front of the singer’s head, it also cannot be excluded that some droplets escaped through the leakages of the masks or moved outside the laser sheet. Thus, the presented numbers do not present the total number of expelled particles but are rather an indicator for the mask filtration ability by comparing the task with and without mask.

Furthermore, although the setup for droplet detection allowed to detect droplets down to a size of 10 μm, the size of droplets being smaller than the pixel resolution of 60 μm/pixel, could not be determined.

## Summary and conclusions

In this work, the effects of surgical masks were investigated during professional classical singing with the focus on the droplet and aerosol particle expulsion originating from the respiratory tract. Two different experiments were performed with ten professional singers of the BR choir who sang the “Ode of Joy” of Ludwig van Beethoven’s 9th symphony with and without a surgical mask. In the first experiment, the singers inhaled the vapor of e-cigarettes that consists of aerosol particles before they started to sing. The analysis of the video footage showed that the aerosols escaped from the mask through leakages at the cheeks and the nose. However, the aerosols stayed in the singer’s near-field around and above the head in contrast to singing without mask for which the aerosol cloud spread far in front of the singer with distances of over 1 m. Thus, the masks kept the aerosol particles near the emitter and deviated the aerosol cloud in larger heights above the singer being less critical to be inhaled by other persons. However, this effect is useless without an effective ventilation concept or an automated ventilation system as the aerosols remain still in the environment becoming especially critical in closed rooms after a certain time period.

In the second experiment, the ballistic droplets were detected within a laser light sheet that was located in front of the singer with the ROI immediately at the singer’s mouth. The particles were recorded by a high-speed camera and subsequently detected and counted for the two tasks of singing with and without mask. For singing with mask, the number of droplets largely decreased. Assuming that those droplets were kept by surgical masks, they were prevented from spreading in large distances in front of the emitter.

As conclusion, the surgical mask is an effective tool to reduce the emission of potentially virus-laden particles over large distances with the potential risk to infect other singers or the audience. However, surgical masks are not optimized for singing as they are too small to tightly cover the singer’s mouth owing to his/her excessive articulation typical for professional singing. Nevertheless, these masks will certainly be an option in layman singing, e.g., in churches and layman choirs.

## Supplementary Information

Supplementary Information

## References

[1] Baka A, Cenciarelli O, Finch E, Karki T, Kinross P, Plachouras D, et al. European Centre for Disease Prevention and Control. Infection prevention and control for COVID-19 in healthcare settings – Third update. 13 May 2020. ECDC: Stockholm; 2020.

[2] Prather KA, Wang CC, Schooley RT (2020). Reducing transmission of SARS-CoV-2. Science.

[3] Zhou M, Zou J (2021). A dynamical overview of droplets in the transmission of respiratory infectious diseases. Phys Fluids.

[4] Asadi S, Wexler AS, Cappa CD, Barreda S, Bouvier NM, Ristenpart WD (2019). Aerosol emission and superemission during human speech increase with voice loudness. Sci Rep.

[5] Correia G, Rodrigues L, da Silva MG, Goncalves T (2020). Airborne route and bad use of ventilation systems as non-negligible factors in SARS-CoV-2 transmission. Med Hypotheses.

[6] Morawska L, Cao J (2020). Airborne transmission of SARS-CoV-2: the world should face the reality. Environ Int.

[7] Setti L, Passarini F, De Gennaro G, Barbieri P, Perrone MG, Borelli M (2020). Airborne transmission route of COVID-19: why 2 meters/6 feet of inter-personal distance could not be enough. Int J Environ Res Public Health.

[8] Hamner L, Dubbel P, Capron I, Ross A, Jordan A, Lee J, et al. High SARS-CoV-2 attack rate following exposure at a choir practice—Skagit County, Washington, March 2020. 2020;69:606–10. MMWR Morb Mortal Wkly Rep. 10.15585/mmwr.mm6919e6 32407303

[9] Van Der Lint P. Die ene Passion die wel doorging, met rampzalige gevolgen. Trouw. 2020.

[10] Stäbler M. Musik in der Corona-Krise: Sollten Chöre wieder gemeinsam singen? Neue Zürcher Zeitung. 2020.

[11] Morawska L, Johnson GR, Ristovski ZD, Hargreaves M, Mengersen K, Corbett S (2009). Size distribution and sites of origin of droplets expelled from the human respiratory tract during expiratory activities. J Aerosol Sci.

[12] Asadi S, Wexler AS, Cappa CD, Barreda S, Bouvier NM, Ristenpart WD (2020). Effect of voicing and articulation manner on aerosol particle emission during human speech. PloS ONE.

[13] Mürbe D, Kriegel M, Lange J, Rotheudt H, Fleischer M (2021). Aerosol emission in professional singing of classical music. Sci Rep.

[14] Mürbe D, Kriegel M, Lange J, Schumann L, Hartmann A, Fleischer M (2020). Aerosol emission of adolescents voices during speaking, singing and shouting. Plos ONE.

[15] Gregson F, Watson NA, Orton CM, Haddrell AE, McCarthy LP, Finnie TJR (2021). Comparing aerosol concentrations and particle size distributions generated by singing, speaking and breathing. Aerosol Sci Technol.

[16] Echternach M, Gantner S, Peters G, Westphalen C, Benthaus T, Jakubaß B (2020). Impulse dispersion of aerosols during singing and speaking: a potential COVID-19 transmission pathway. Am J Respir Crit Care Med.

[17] Bayern KK. Schutzkonzept der bayerischen (Erz-) Diazösen nach Abstimmung mit der Bayerischen Staatsregierung: Rahmenbedingungen und möglicher Ablauf Gottesdienst mit beschränkter Teilnehmerzahl online. 04.10.2021. 2020. https://information.erzbistum-bamberg.de/medien/55a9bff1-f1a7-448f-85e9-406e8e27a3c3/200501_schutzkonzept_der_bayerischen_dioezesen.pdf?a=true.

[18] Klingan C. Infektionsschutzkonzept für katholische Gottesdienste im Erzbistum München und Freising online. 11.03.2021. 2020. https://www.erzbistum-muenchen.

[19] Regmi K, Lwin CM (2021). Factors associated with the implementation of non-pharmaceutical interventions for reducing coronavirus disease 2019 (COVID-19): a systematic review. Int J Environ Res Public Health.

[20] Chu D, Akl EA, Duda S, Solo K, Yaacoub S, Schünemann HJ (2020). Physical distancing, face masks, and eye protection to prevent person-to-person transmission of SARS-CoV-2 and COVID-19: a systematic review and meta-analysis. Lancet.

[21] Asadi S, Cappa CD, Barreda S, Wexler AS, Bouvier NM, Ristenpart WD (2020). Efficacy of masks and face coverings in controlling outward aerosol particle emission from expiratory activities. Sci Rep.

[22] Ueki H, Furusawa Y, Iwatsuki-Horimoto K, Imai M, Kabata H, Nishimura H (2020). Effectiveness of face masks in preventing airborne transmission of SARS-CoV-2. mSphere.

[23] Verma S, Dhanak M, Frankenfield J (2020). Visualizing the effectiveness of face masks in obstructing respiratory jets. Phys Fluids.

[24] Ishii K, Ohno Y, Oikawa M, Onishi N (2021). Relationship between human exhalation diffusion and posture in face-to-face scenario with utterance. Phys Fluids.

[25] Lei Z, Yang J, Zhuang Z, Roberge R (2013). Simulation and evaluation of respirator faceseal leaks using computational fluid dynamics and infrared imaging. Ann Occup Hyg.

[26] Dbouk T, Drikakis D (2020). On respiratory droplets and face masks. Phys Fluids.

[27] Tcharkhtchi A, Abbasnezhad N, Zarbini Seydani M, Zirak N, Farzaneh S, Shirinbayan M (2021). An overview of filtration efficiency through the masks: Mechanisms of the aerosols penetration. Bioact Mater.

[28] Sharma S, Pinto R, Saha A, Chaudhuri S, Basu S (2021). On secondary atomization and blockage of surrogate cough droplets in single- and multilayer face masks. Sci Adv.

[29] Bourouiba L (2020). Turbulent gas clouds and respiratory pathogen emissions: potential implications for reducing transmission of COVID-19. JAMA.

[30] Tellier R, Li Y, Cowling B, Tang J (2019). Recognition of aerosol transmission of infectious agents: a commentary. BMC Infect Dis.

[31] Bourouiba L, Dehandschoewercker E, John WB (2014). Violent expiratory events: on coughing and sneezing. J Fluid Mech.

[32] Kutter J, Spronken M, Fraaij P, Fouchier R, Herfst S (2018). Transmission routes of respiratory viruses among humans. Curr Opin Virol.

[33] Kähler CJ, Hain R (2020). Fundamental protective mechanisms of face masks against droplet infections. J Aerosol Sci.

[34] Merghani JMM, Sagot B, Gehin E, Da G, Motzkus C (2020). A review on the applied techniques of exhaled airflow and droplets characterization. Indoor Air.

[35] Wang H, Li Z, Zhang X, Zhu L, Liu Y, Wang S (2020). The motion of respiratory droplets produced by coughing. Phys Fluids.

[36] Gena AW, Voelker C, Settles GS (2020). Qualitative and quantitative schlieren optical measurement of the human thermal plume. Indoor Air.

[37] Tan ZP, Silwal L, Bhatt SP, Raghav V (2021). Experimental characterization of speech aerosol dispersion dynamics. Sci Rep.

[38] Crawford C, Vanoli E, Decorde B, Lancelot M, Duprat C, Josserand C (2021). Modeling of aerosol transmission of airborne pathogens in ICU rooms of COVID-19 patients with acute respiratory failure. Sci Rep.

[39] Arumuru V, Pasa J, Samantaray SS (2020). Experimental visualization of sneezing and efficacy of face masks and shields. Phys Fluids.

[40] Gantner S, Caffier P, Hulin P, Kummer P, Lorenz A. Entwicklung und Validierung des Singing Voice Handicap Index-12 in 36. Wissenschaftliche Jahrestagung der Deutschen Gesellschaft für Phoniatrie und Pädaudiologie (DGPP), 19.09.—22.09.2019, Göttingen (Deutsche Gesellschaft für Phoniatrie und Pädaudiologie e. V., 2019).

[41] Gantner S, Echternach M, Veltrup R, Westphalen C, Köberlein MC, Kuranova L, et al. Impulse dispersion of aerosols during playing wind instruments. medRxiv. 2021. 10.1101/2021.01.25.20248984. 10.1371/journal.pone.0262994 PMC8893631 35239657

[42] Staymates M (2020). Flow visualization of an N95 respirator with and without an exhalation valve using schlieren imaging and light scattering. Phys Fluids.

[43] Giovanni A, Radulesco T, Bouchet G, Mattei A, Révis J, Bogdanski E (2021). Transmission of droplet-conveyed infectious agents such as SARS-CoV-2 by speech and vocal exercises during speech therapy: preliminary experiment concerning airflow velocity. Eur Arch Otorhinolaryngol.

[44] Ingebrethsen BJ, Cole SK, Alderman SL (2012). Electronic cigarette aerosol particle size distribution measurements. Inhal Toxicol.

[45] Bandiera L, Pavar G, Pisetta G, Otomo S, Mangano E, Seckl JR (2020). Face coverings and respiratory tract droplet dispersion. R Soc Open Sci.

[46] Lodermeyer A, Tautz M, Becker S, Döllinger M, Birk V, Kniesburges S (2018). Aeroacoustic analysis of the human phonation process based on a hybrid acoustic PIV approach. Exp Fluids.

[47] Westphalen C, Kniesburges S, Veltrup R, Gantner S, Peters G, Benthaus T, et al. Sources of aerosol dispersion during singing and potential safety procedures for singers. J Voice. 2021. In press. 10.1016/j.jvoice.2021.03.013 33849763

[48] Jarvis M (2020). Aerosol transmission of SARS-CoV-2: physical principles and implications. Front Public Health.

[49] Alsved M, Matamis A, Bohlin R, Richter M, Bengtsson P-E, Fraenkel C-J (2020). Exhaled respiratory particles during singing and talking. Aerosol Sci Technol.

[50] Tang JW, Liebner TJ, Craven BA, Settles GS (2009). A schlieren optical study of the human cough with and without wearing masks for aerosol infection control. J R Soc Interface.

[51] Mitze T, Kosfeld R, Rode J, Wälde K (2020). Face masks considerably reduce COVID- 19 cases in Germany. Proc Natl Acad Sci USA.

[52] Lieber C, Melekidis S, Koch R, Bauer H-J (2021). Insights into the evaporation characteristics of saliva droplets and aerosols: levitation experiments and numerical modeling. J Aerosol Sci.

[53] Kolewe E, Stillman Z, Woodward I, Fromen C (2020). Check the gap: facemask performance and exhaled aerosol distributions around the wearer. PloS ONE.

[54] Li L, Niu M, Zhu Y (2021). Assessing the effectiveness of using various face coverings to mitigate the transport of airborne particles produced by coughing indoors. Aerosol Sci Technol.

[55] Hariharan P, Sharma N, Guha S, Banerjee RK, D’Souza G, Myers MR (2021). A computational model for predicting changes in infection dynamics due to leakage through N95 respirators. Sci Rep.

[56] Zhu JH, Lee SJ, Wang DY, Lee HP (2016). Evaluation of rebreathed air in human nasal cavity with N95 respirator: a CFD study. Trauma Emerg Care.

[57] Tejedor B, Casals M, Gangolells M, Macarulla M, Forcada N (2020). Human comfort modelling for elderly people by infrared thermography: Evaluating the thermoregulation system responses in an indoor environment during winter. Build Environ.

