# Supplementary material for: Effects of surgical masks on aerosol dispersion in professional singing
Source: J Expo Sci Environ Epidemiol. 2021 Oct 5;32(5):727–34. doi: 10.1038/s41370-021-00385-7 (PMC8491963; doi:10.1038/s41370-021-00385-7)
Supplement: Supplementary file 1 — Supplementary Information [file 41370_2021_385_MOESM1_ESM.pdf]

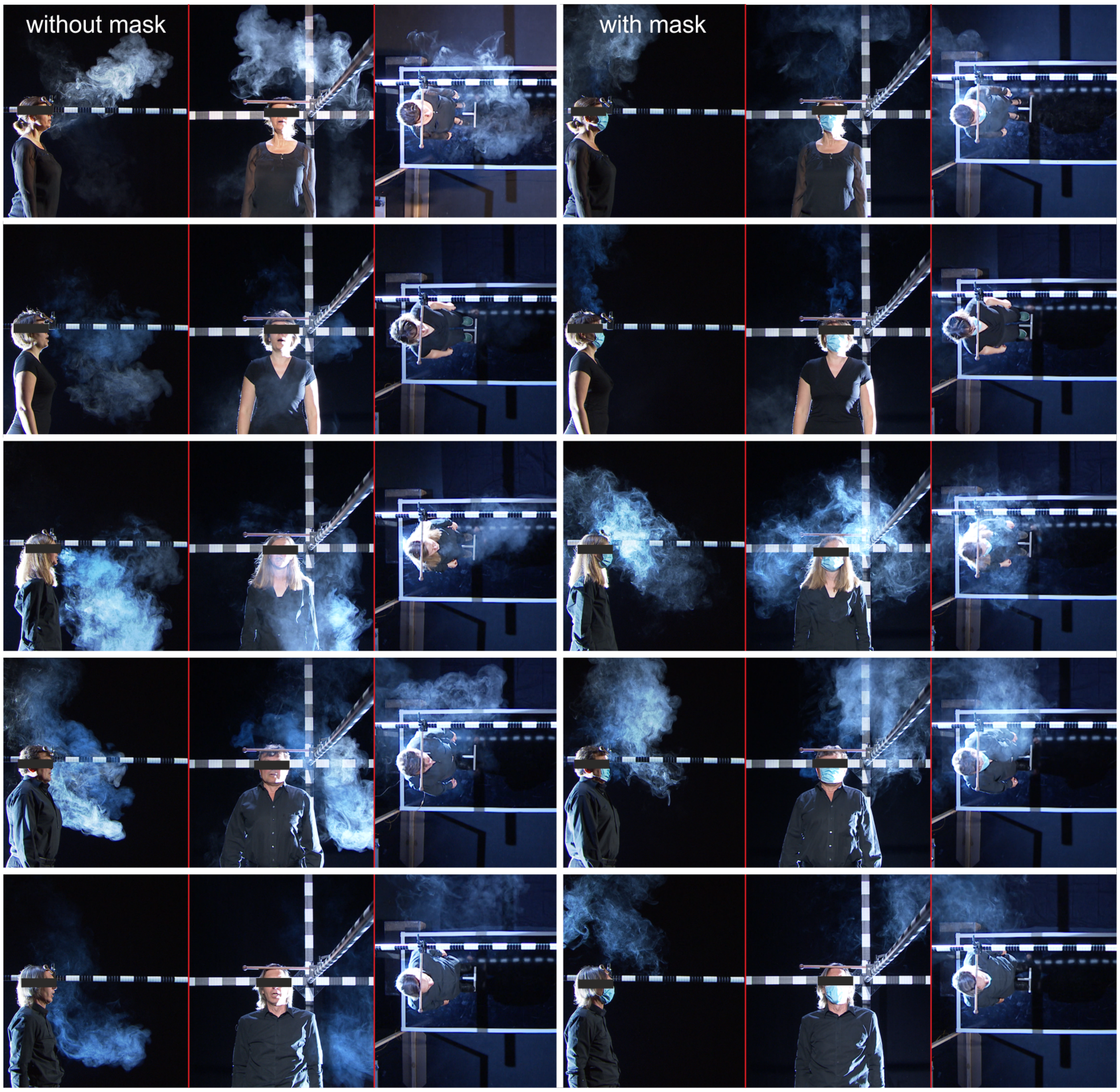

Supplement Figure 1: Visualization of the aerosol emitted by the singers #1 - #5 during singing with (right) and without mask (left). The pictures were kept from the video footage at the end of the task in the side, front and top perspective.

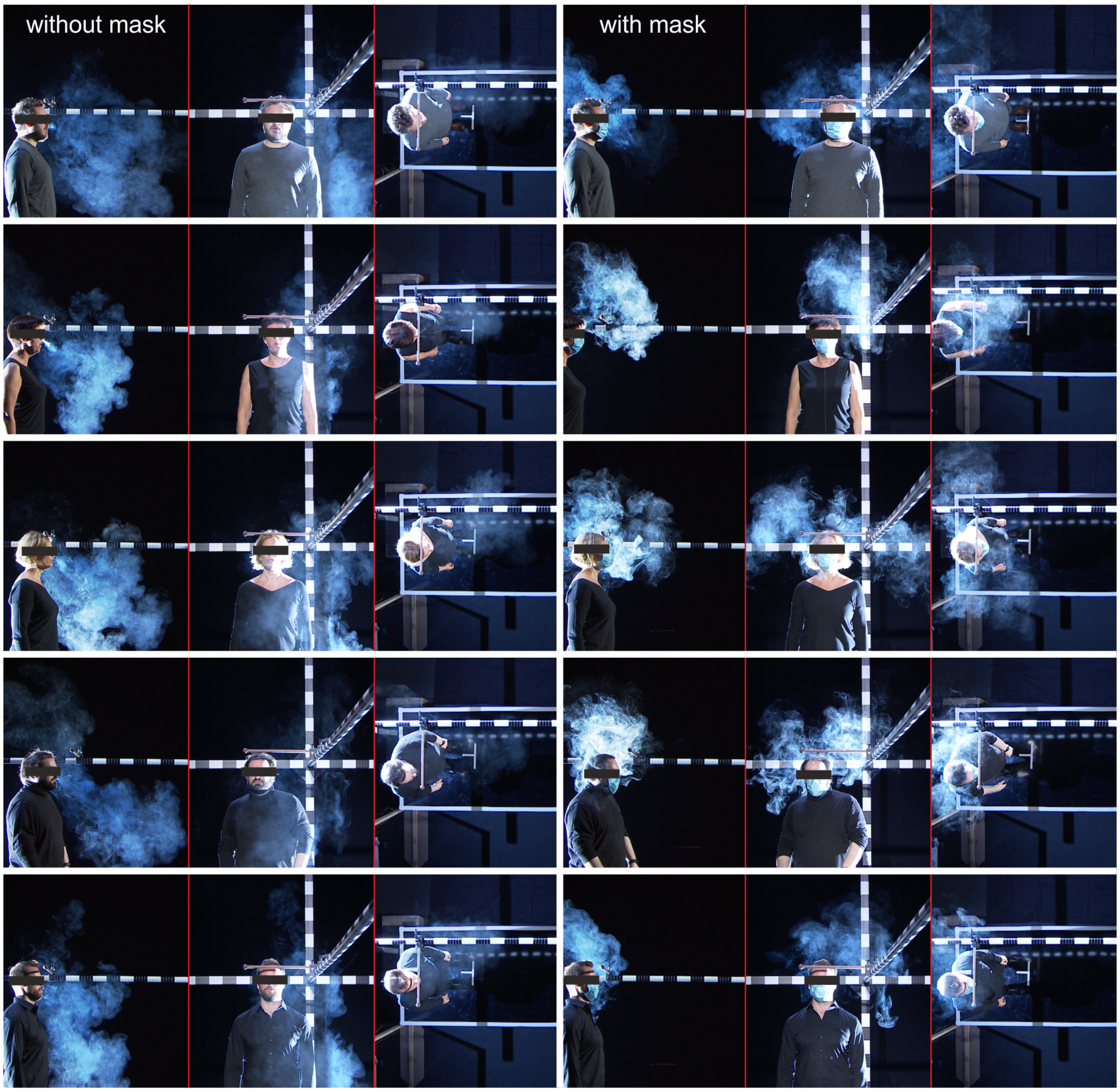

Supplement Figure 2: Visualization of the aerosol emitted by singers #6 - #10 during singing with (right) and without mask (left). The pictures were kept from the video footage at the end of the task in the side, front and top perspective.
